# Supplementary material for: Detection of acute dengue virus infection, with and without concurrent malaria infection, in a cohort of febrile children in Kenya, 2014–2019, by clinicians or machine learning algorithms
Source: PLOS Glob Public Health. 2023 Jul 26;3(7):e0001950. doi: 10.1371/journal.pgph.0001950 (PMC10370704; doi:10.1371/journal.pgph.0001950)
Supplement: S1 Table — (DOCX) [file pgph.0001950.s002.docx]

S1 Table. Characteristics of all enrolled subjects with acute febrile illness^a^ by region or urban/rural

|  | Total | By region | | *p^b^* (SMD^c^) | By urban or rural | | *p^b^* (SMD^c^) |
| --- | --- | --- | --- | --- | --- | --- | --- |
|  |  | west | coast |  | urban | rural |  |
| Enrolled, n | 7,509 | 2,544 | 4,965 |  | 4,268 | 3,241 |  |
| Female, n (%) | 3,585 (47.7) | 1,210 (47.6) | 2,375 (47.8) | 0.84 (NA) | 2,039 (47.8) | 1,549 (47.7) | 0.97 (NA) |
| Age, median years (IQR) | 4.8 (2.9, 8.4) | 3.7 (2.2, 5.8) | 5.5 (3.3, 9.7) | <0.001 (0.60) | 5.5 (3.3, 9.3) | 4.1 (2.4, 6.7) | <0.001 (0.34) |
| Height-for-age, median z (IQR) | -0.8 (-1.7, 0.2) | -0.6 (-1.6, 0.3) | -0.8 (-1.8, 0.1) | <0.001 (0.13) | -0.7 (-1.6,0.2) | -0.9 (-1.8, 0.1) | <0.001 (0.09) |
| BMI-for-age, median z (IQR) | -0.7 (-1.5, 0.2) | -0.3 (-1.1, 0.6) | -0.9 (-1.7, -0.1) | <0.001 (0.44) | -0.7 (-1.7, 0.2) | -0.6 (-1.5, 0.2) | 0.003 (0.06) |
| Wealth index, median (IQR) | 2 (1, 3) | 3 (2, 3) | 2 (1, 3) | <0.001 (0.39) | 2 (2, 3) | 2 (1, 3) | <0.001 (0.33) |

^a^ Acute febrile illness defined as report of fever as symptom or recorded temperature ≥38 degrees Celsius at the initial clinic visit

^b^ Categorical variables tested by chi-square, and continuous variable tested using the Kruskal-Wallis H test

^c^ Standard mean differences (SMD) available for continuous variables only

Abbreviations: SMD, standard mean difference; IQR, interquartile range; BMI, body mass index
