# Supplementary material for: Detection of acute dengue virus infection, with and without concurrent malaria infection, in a cohort of febrile children in Kenya, 2014–2019, by clinicians or machine learning algorithms
Source: PLOS Glob Public Health. 2023 Jul 26;3(7):e0001950. doi: 10.1371/journal.pgph.0001950 (PMC10370704; doi:10.1371/journal.pgph.0001950)
Supplement: S2 Table — (DOCX) [file pgph.0001950.s003.docx]

S2 Table. Characteristics of all enrolled and complete cases^a^ cohort

|  | Enrolled | Excluded | Complete cases | *p^b^* | SMD^c^ |
| --- | --- | --- | --- | --- | --- |
| Total enrolled, n (%) | 7,509 | 1301 | 6208 |  |  |
| Female, n (%) | 3,585 (47.7) | 594 (45.7) | 2991 (48.2) | 0.10 | NA |
| Age, median years (IQR) | 4.8 (2.9, 8.4) | 5 (2.9, 9.0) | 4.8 (2.9, 8.3) | 0.03 | 0.090 |
| Height-for-age, median z-score (IQR) | -0.8 (-1.7, 0.2) | -0.7 (-1.7, 0.3) | -0.8 (-1.7, 0.1) | 0.34 | 0.037 |
| BMI-for-age, median z-score (IQR) | -0.7 (-1.6, 0.2) | -0.8 (-1.7, 0.1) | -0.7 (-1.5, 0.2) | 0.004 | 0.088 |
| Wealth index, median (IQR) | 2 (1, 3) | 2 (1, 3) | 2 (1, 3) | <0.001 | 0.17 |
| DENV positive, n (%) | 490 (7.9) | 5 (17.8) | 485 (7.8) | 0.11 | NA |
| Malaria positive, n (%) | 3,757 (50.2) | 612 (47.0) | 3145 (50.7) | 0.10 | NA |
| Referred for hospital admission, n (%) | 247 (3.3) | 32 (2.5) | 215 (3.5) | 0.26 | NA |

^a^Complete cases were defined as having both malaria and DENV test results

^b^Categorical variables tested by chi-square, and continuous variable tested using the Kruskal-Wallis H test

^c^Standard mean differences available for continuous variables only

Abbreviations: SMD, standard mean difference; IQR, interquartile range; BMI, body mass index; DENV, dengue virus
