# Supplementary material for: Detection of acute dengue virus infection, with and without concurrent malaria infection, in a cohort of febrile children in Kenya, 2014–2019, by clinicians or machine learning algorithms
Source: PLOS Glob Public Health. 2023 Jul 26;3(7):e0001950. doi: 10.1371/journal.pgph.0001950 (PMC10370704; doi:10.1371/journal.pgph.0001950)
Supplement: S3 Table — (DOCX) [file pgph.0001950.s004.docx]

S3 Table. Supporting statistics for Table 2.

|  | Chi square statistic | df | *p* | DENV/malaria co-infection | DENV solo-infection | malaria solo-infection | DENV/malaria uninfected |
| --- | --- | --- | --- | --- | --- | --- | --- |
| By sex | 2.61 | 3 | 0.46 |  |  |  |  |
| Female, residual (% contribution) |  |  |  | -0.41 (6.6) | -0.87 (29.2) | 0.60 (13.9) | -0.24 (2.1) |
| Male, residual (% contribution) |  |  |  | 0.40 (6.1) | 0.84 (27.2) | -0.58 (12.9) | 0.23 (2.0) |
| By admission to hospital | 38.2 | 3 | <0.001 |  |  |  |  |
| \| Admitted, residual (% contribution) \| \| --- \| |  |  |  | 2.18 (12.4) | -0.25 (0.2) | 3.72 (36.2) | -4.17 (45.5) |
| \| Not admitted, residual (% contribution) \| \| --- \| |  |  |  | -0.54 (0.8) | 0.06 (0.01) | -0.92 (2.2) | 1.03 (2.8) |
| By study site | 686 | 9 | <0.001 |  |  |  |  |
| Kisumu, residual (% contribution) |  |  |  | 1.63 (0.4) | 6.71 (6.6) | -4.74 (3.3) | 2.4 (0.8) |
| Chulaimbo, residual (% contribution) |  |  |  | 15.20 (33.7) | 0.52 (0.04) | 8.51 (10.6) | -13.4 (26.0) |
| Ukunda, residual (% contribution) |  |  |  | -6.51 (6.2) | -4.95 (3.6) | 2.02 (0.6) | 1.33 (0.3) |
| Msambweni, residual (% contribution) |  |  |  | -4.41 (2.8) | -1.21 (0.2) | -3.23 (1.5) | 4.94 (3.6) |
| By geography | 270 | 3 | <0.001 |  |  |  |  |
| West, residual (% contribution) |  |  |  | 10.18 (38.3) | 5.76 (12.2) | 1.10 (0.5) | -5.82 (12.5) |
| Coast, residual (% contribution) |  |  |  | -7.73 (22.0) | -4.37 (7.1) | -0.84 (0.3) | 4.42 (7.2) |
| By rural/urban | 55.7 | 3 | <0.001 |  |  |  |  |
| Urban, residual (% contribution) |  |  |  | -3.85 (26.6) | 0.66 (0.8) | -1.58 (4.5) | 2.58 (12.0) |
| Rural, residual (% contribution) |  |  |  | 4.36 (34.1) | -0.75 (1.0) | 1.79 (5.8) | -2.92 (15.4) |
