# Supplementary material for: Detection of acute dengue virus infection, with and without concurrent malaria infection, in a cohort of febrile children in Kenya, 2014–2019, by clinicians or machine learning algorithms
Source: PLOS Glob Public Health. 2023 Jul 26;3(7):e0001950. doi: 10.1371/journal.pgph.0001950 (PMC10370704; doi:10.1371/journal.pgph.0001950)
Supplement: S4 Table — (DOCX) [file pgph.0001950.s005.docx]

S4 Table. Supporting statistics for Table 3.

|  | Chi square statistic | *p* | Chi square residual (% contribution) | | | |
| --- | --- | --- | --- | --- | --- | --- |
| Clinical feature |  |  | DENV/malaria co-infection | DENV solo-infection | Malaria solo-infection | DENV/malaria uninfected |
| Chills ^S^ | 82.2 | <0.001 |  |  |  |  |
| Present |  |  | -0.18 (0.04) | -1.76 (3.8) | 5.75 (40.3) | -5.25 (33.5) |
| Absent |  |  | 0.10 (0.01) | 0.94 (1.1) | -3.09 (11.6) | 2.82 (9.7) |
| Malaise ^S^ | 36.2 | <0.001 |  |  |  |  |
| Present |  |  | -0.86 (2.0) | 1.25 (4.3) | 2.98 (24.6) | -3.09 (26.4) |
| Absent |  |  | 0.74 (1.5) | -1.08 (3.2) | -2.58 (18.3) | 2.67 (19.6) |
| Aches and pains ^S^ | 65.2 | <0.001 |  |  |  |  |
| Present |  |  | -0.66 (0.7) | -0.51 (0.4) | 4.95 (37.7) | -4.64 (33.1) |
| Absent |  |  | 0.26 (0.4) | 0.16 (0.3) | -3.10 (14.8) | 2.91 (13.0) |
| Loss of appetite ^S^ | 46.8 | <0.001 |  |  |  |  |
| Present |  |  | 1.59 (5.4) | 2.70 (15.6) | 2.56 (14.0) | -3.81 (31.1) |
| Absent |  |  | -1.14 (2.8) | -1.94 (8.0) | -1.83 (7.2) | 2.73 (16.0) |
| Headaches ^S^ | 49.5 | <0.001 |  |  |  |  |
| Present |  |  | 0.09 (0.02) | 0.09 (0.02) | 3.53 (25.1) | -3.60 (26.2) |
| Absent |  |  | -0.09 (0.01) | -0.09 (0.01) | -3.43 (23.8) | 3.51 (24.8) |
| Dizziness ^S^ | 62.7 | <0.001 |  |  |  |  |
| Present |  |  | -2.64 (11.1) | -1.61 (4.1) | 5.57 (49.4) | -4.35 (30.2) |
| Absent |  |  | 0.62 (0.6) | 0.38 (0.2) | -1.30 (2.7) | 1.02 (1.7) |
| Altered behavior ^S^ | 28.7 | <0.001 |  |  |  |  |
| Present |  |  | 4.92 (84.2) | -0.92 (3.0) | 0.56 (1.1) | -1.81 (11.4) |
| Absent |  |  | -0.31 (0.3) | 0.06 (0.01) | -0.03 (0) | 0.11 (0.04) |
| Lethargic ^O^ | 18.3 | <0.001 |  |  |  |  |
| Present |  |  | 1.91 (20.0) | 2.34 (29.8) | 0.88 (4.2) | -2.12 (24.6) |
| Absent |  |  | -1.0 (5.4) | -1.22 (8.1) | -0.46 (1.2) | 1.11 (6.7) |
| Weakness ^O^ | 60.2 | <0.001 |  |  |  |  |
| Present |  |  | 5.90 (57.9) | 4.50 (33.7) | -1.84 (5.6) | -1.2 (2.4) |
| Absent |  |  | -0.37 (0.2) | -0.28 (0.1) | 0.11 (0.02) | 0.07 (0.01) |
| Coryza ^S^ | 27.8 | <0.001 |  |  |  |  |
| Present |  |  | -1.74 (10.9) | -1.59 (9.1) | -2.78 (27.8) | 3.77 (51.1) |
| Absent |  |  | 0.19 (0.1) | 0.17 (0.1) | 0.30 (0.3) | -0.41 (0.6) |
| Conjunctival injection ^O^ | 55.9 | <0.001 |  |  |  |  |
| Present |  |  | 2.57 (11.9) | 6.61 (78.2) | -1.87 (6.3) | -0.74 (1.0) |
| Absent |  |  | -0.43 (0.3) | -1.10 (2.2) | 0.31 (0.2) | 0.12 (0.03) |

S4 Table S4 (continued). Supporting statistics for Table 3.

|  | Chi square statistic | *p* | Chi square residual (% contribution) | | | |
| --- | --- | --- | --- | --- | --- | --- |
| Clinical feature |  |  | DENV/malaria co-infection | DENV solo-infection | Malaria solo-infection | DENV/malaria uninfected |
| Runny nose ^S^ | 113 | <0.001 |  |  |  |  |
| Present |  |  | -0.23 (0.1) | 2.11 (3.9) | -6.46 (37.1) | 5.99 (31.8) |
| Absent |  |  | 0.14 (0.02) | -1.29 (1.5) | 3.94 (13.8) | -3.65 (11.9) |
| Sore throat ^S^ | 66.8 | <0.001 |  |  |  |  |
| Present |  |  | -2.1 (6.3) | -1.3 (2.6) | -4.86 (35.4) | 5.88 (51.8) |
| Absent |  |  | 0.42 (0.3) | 0.27 (0.1) | 1.0 (1.5) | -1.21 (2.2) |
| Cough ^S^ | 96.3 | <0.001 |  |  |  |  |
| Present |  |  | 0.58 (0.4) | 1.53 (2.4) | -5.47 (31.1) | 4.91 (25.0) |
| Absent |  |  | -0.48 (0.2) | -1.28 (1.7) | 4.57 (21.7) | -4.10 (17.5) |
| Ronchi or rales ^O^ | 19.0 | <0.001 |  |  |  |  |
| Present |  |  | -0.67 (2.4) | -1.02 (5.4) | -2.68 (37.7) | 3.18 (53.3) |
| Absent |  |  | 0.07 (0.03) | 0.11 (0.07) | 0.30 (0.5) | -0.35 (0.7) |
| Tachycardia ^O^ | 50.8 | <0.001 |  |  |  |  |
| Present |  |  | 0.93 (1.7) | 0.03 (0) | 4.54 (40.5) | -4.86 (46.5) |
| Absent |  |  | -0.33 (0.2) | -0.01 (0) | -1.62 (5.2) | 1.73 (5.9) |
| Abdominal pain ^S^ | 103.6 | <0.001 |  |  |  |  |
| Present |  |  | -2.38 (5.5) | -3.96 (15.1) | 6.15 (36.5) | -4.36 (18.4) |
| Absent |  |  | 1.36 (1.8) | 2.26 (4.9) | -3.51 (11.9) | 2.49 (6.0) |
| Nausea ^S^ | 21.1 | <0.001 |  |  |  |  |
| Present |  |  | 0.61 (1.8) | -0.92 (4.0) | 3.07 (44.6) | -3.02 (43.1) |
| Absent |  |  | -0.16 (0.1) | 0.24 (0.3) | -0.81 (3.1) | 0.80 (3.0) |
| Vomiting ^S^ | 167 | <0.001 |  |  |  |  |
| Present |  |  | 1.72 (1.8) | -1.0 (0.6) | 7.22 (31.3) | -7.51 (33.9) |
| Absent |  |  | -1.19 (0.9) | 0.69 (0.3) | -5.01 (15.0) | 5.21 (16.3) |
| Splenomegaly ^O^ | 17.1 | <0.001 |  |  |  |  |
| Present |  |  | 2.43 (34.7) | 2.77 (45.1) | 0.19 (0.2) | -1.71 (17.2) |
| Absent |  |  | -0.41 (1.0) | -0.47 (1.3) | -0.03 (0.01) | 0.29 (0.5) |
| Myalgia ^S^ | 30.4 | <0.001 |  |  |  |  |
| Present |  |  | 4.3 (62.0) | 1.1 (4.0) | 1.1 (4.2) | -2.76 (25.1) |
| Absent |  |  | -0.97 (3.1) | -0.24 (0.2) | -0.25 (0.2) | 0.62 (1.3) |
| Joint erythema ^O^ | 34.0 | <0.001 |  |  |  |  |
| Present |  |  | 3.69 (39.9) | 4.21 (52.1) | -1.16 (4.0) | -1.13 (3.8) |
| Absent |  |  | -0.20 (0.1) | -0.23 (0.2) | 0.06 (0.01) | 0.06 (0.01) |

S4 Table S4 (continued). Supporting statistics for Table 3.

|  | Chi square statistic | *p* | Chi square residual (% contribution) | | | |
| --- | --- | --- | --- | --- | --- | --- |
| Clinical feature |  |  | DENV/malaria co-infection | DENV solo-infection | Malaria solo-infection | DENV/malaria uninfected |
| Joint tenderness ^O^ | 81.6 | <0.001 |  |  |  |  |
| Present |  |  | 5.46 (36.6) | 6.13 (46.1) | -1.55 (3.0) | -1.81 (4.0) |
| Absent |  |  | -1.86 (4.2) | -2.08 (5.3) | 0.53 (0.3) | 0.61 (0.5) |
| Bones ache ^S^ | 13.7 | <0.001 |  |  |  |  |
| Present |  |  | 2.29 (38.1) | -0.01 (0) | 1.66 (20.2) | -2.37 (40.9) |
| Absent |  |  | -0.22 (0.4) | 0 (0) | -0.16 (0.2) | 0.23 (0.4) |
| Rash ^S^ | 14.5 | <0.001 |  |  |  |  |
| Present |  |  | -1.07 (7.9) | 1.00 (6.8) | -2.42 (40.3) | 2.48 (42.5) |
| Absent |  |  | 0.17 (0.2) | -0.16 (0.2) | 0.39 (1.1) | -0.40 (1.1) |
| Maculopapular exanthem ^O^ | 21.7 | <0.001 |  |  |  |  |
| Present |  |  | -1.90 (16.6) | 0.71 (2.3) | -2.73 (34.2) | 3.13 (44.9) |
| Absent |  |  | 0.27 (0.4) | -0.10 (0.1) | 0.39 (0.7) | -0.45 (0.9) |
| Cervical adenopathy ^O^ | 56.5 | <0.001 |  |  |  |  |
| Present |  |  | -0.08 (0.01) | 4.00 (28.3) | -4.57 (36.9) | 3.51 (21.8) |
| Absent |  |  | 0.03 (0) | -1.55 (4.2) | 1.77 (5.5) | -1.36 (3.3) |

^S^: reported symptom

^O^: observed physical exam finding
