# Supplementary material for: Detection of acute dengue virus infection, with and without concurrent malaria infection, in a cohort of febrile children in Kenya, 2014–2019, by clinicians or machine learning algorithms
Source: PLOS Glob Public Health. 2023 Jul 26;3(7):e0001950. doi: 10.1371/journal.pgph.0001950 (PMC10370704; doi:10.1371/journal.pgph.0001950)
Supplement: S5 Table — (DOCX) [file pgph.0001950.s006.docx]

S5 Table. Performance characteristics for predicting absence of DENV and absence of malaria infection in the validation dataset

| Laboratory diagnosis (n=1861) | TP (n=852) | FN | FP | TN  (n=1009) | Acc | Sens | Spec | PPV | NPV | Kappa | Tuning  parameter |
| --- | --- | --- | --- | --- | --- | --- | --- | --- | --- | --- | --- |
| Clinician diagnosis | 453 | 399 | 142 | 867 | 0.71 | 0.53 | 0.86 | 0.76 | 0.68 | 0.40 | NA |
| Boosted logistic regression | 629 | 465 | 176 | 474 | 0.60 | 0.58 | 0.73 | 0.78 | 0.50 | 0.25 | nIter = 21 |
| Classification tree | 628 | 464 | 545 | 224 | 0.59 | 0.58 | 0.71 | 0.74 | 0.54 | 0.24 | Cp=0.0163 |
| Random forest | 609 | 353 | 646 | 240 | 0.63 | 0.63 | 0.73 | 0.71 | 0.65 | 0.32 | Mtry=71 |
| Support vector machines | 638 | 401 | 608 | 214 | 0.63 | 0.61 | 0.74 | 0.75 | 0.61 | 0.31 | Sigma=0.0241, C=1 |
| Naïve Bayes | 852 | 0 | 1009 | 0 | 0.46 | 0.46 | NA | NA | NA | 0 | Laplace=0, Usekernel=T, Adjust=1 |
| Neural networks (MLP) | 555 | 681 | 328 | 297 | 0.61 | 0.63 | 0.70 | 0.65 | 0.67 | 0.28 | Size=5, decay=0 |

Abbreviations: TP, true positive; FN, false negative; FP, false positive; TN, true negative; Acc, accuracy; Sens, sensitivity; Spec, specificity; PPV, positive predictive value; NPV, negative predictive value; MLP, multi-layer perceptron; nIter, number of iterations; Cp, complexity parameter; Mtry, number of trees
