# Supplementary material for: Detection of acute dengue virus infection, with and without concurrent malaria infection, in a cohort of febrile children in Kenya, 2014–2019, by clinicians or machine learning algorithms
Source: PLOS Glob Public Health. 2023 Jul 26;3(7):e0001950. doi: 10.1371/journal.pgph.0001950 (PMC10370704; doi:10.1371/journal.pgph.0001950)
Supplement: S1 Text — (DOCX) [file pgph.0001950.s007.docx]

| **Demography Survey** |  |
| --- | --- |
| Person ID |  |
| Are demography data available? | ○ Yes  ○ No |
| StudyID HCC |  |
| StudyID Demography Data |  |
| Demography Interview Date |  |
| Village | ○ Chulaimbo  ○ Kisumu  ○ Msambweni  ○ Ukunda  ○ N/A |
| Other Village |  |
| Interviewer's name |  |
| Specify other interviewer's name |  |
| Lives in a compound? | ○ Yes  ○ No  ○ Refused |
| Compound number |  |
| **Head of Compound** |  |
| Head of compound StudyID |  |
| Head of compound gender | ○ Female  ○ Male  ○ Refused |
| Head of compound age (years) |  |
| Head of compound category | ○ Child  ○ Junior  ○ Adult  ○ Other  ○ Refused |
| Head of compound language | ○ Swahili  ○ Duruma  ○ Digo  ○ kamba Luo  ○ Luhya  ○ Kisii  ○ Nubian  ○ Kalenjin  ○ Nandi  ○ Kuria  ○ Kikuyu  ○ Maasai  ○ Buganda  ○ Other  ○ Refused |

| Head of compound other language |  |
| --- | --- |
| Head of compound tribe | ○ Digo  ○ Duruma  ○ kamba Luo  ○ Luhya  ○ Kisii  ○ Nubian  ○ Kalenjin  ○ Nandi  ○ Kuria  ○ Kikuyu  ○ Maasai  ○ Buganda  ○ Other  ○ Refused |
| Head of compound other tribe |  |
| Head of compound religion | ○ Islam/Muslim  ○ Christianity  ○ Other  ○ Refused |
| Head of compound other religion |  |
| Is the head of compound married | ○ No  ○ Yes  ○ Divorced  ○ Widowed  ○ Separated  ○ Inherited  ○ Other  ○ Refused |
| Head of compound other married |  |
| Does the head of compound sleep in the house? | ○ Yes  ○ No  ○ Refused |
| House number |  |
| House pic |  |
| **Head of Household** |  |
| Head of household StudyID |  |
| Head of household gender | ○ Female  ○ Male  ○ Refused |
| Head of household age (years) |  |
| Head of household category | ○ Child  ○ Junior  ○ Adult  ○ Other  ○ Refused |
| Head of household language | ○ Swahili  ○ Duruma  ○ Digo  ○ kamba Luo  ○ Luhya  ○ Kisii  ○ Nubian  ○ Kalenjin  ○ Nandi  ○ Kuria  ○ Kikuyu  ○ Maasai  ○ Buganda  ○ Other  ○ Refused |

| Head of household other language |  |
| --- | --- |
| Head of household tribe | ○ Digo  ○ Duruma  ○ kamba Luo  ○ Luhya  ○ Kisii  ○ Nubian  ○ Kalenjin  ○ Nandi  ○ Kuria  ○ Kikuyu  ○ Maasai  ○ Buganda  ○ Other  ○ Refused |
| Head of household other tribe |  |
| Head of household religion | ○ Islam/Muslim  ○ Christianity  ○ Other  ○ Refused |
| Head of household other religion |  |
| Is the head of household married | ○ No  ○ Yes  ○ Divorced  ○ Widowed  ○ Separated  ○ Inherited  ○ Other  ○ Refused |
| Head of household other married |  |
| Does the head of household have children? | ○ Yes  ○ No  ○ Refused |
| Number of children |  |
| Type of house ownership | ○ Own  ○ Rent  ○ Other  ○ Refused |
| Head of household other house |  |
| Does the head of household sleep in the house? | ○ Yes  ○ No  ○ Refused |
| Does the head of household live in the house? | ○ Yes  ○ No  ○ Refused |
| Years head of household has lived in district |  |
| Years head of household has lived in house |  |
| Does the head of household sleep in a bedroom? |  |
| People per room in head of household’s room |  |
| Number of windows in head of household’s room |  |
| Do windows in head of household’s room have screens | ○ Yes  ○ No  ○ Refused |
| Do the people in the head of household’s room sleep with the windows closed? | ○ Yes  ○ No  ○ Refused |
| Does the head of household own a bednet? | ○ Yes  ○ No  ○ Refused |
| Number bednets owned by head of household? |  |
| Does the head of household sleep under a bednet | ○ Yes  ○ No  ○ Refused |
| Doe the kids of the head of household sleep under a bednet | ○ All  ○ None  ○ Some  ○ Refused |
| What does the head of household use for mosquito control |  |
| Does the head of household own a communal tv | ○ Yes  ○ No  ○ Refused |
| What methods does the head of household use for water collection |  |
| Which other? |  |
| What is the material of the head of household’s floor? | ○ Dirt/earth  ○ Wood plank  ○ Tile  ○ Cement  ○ Other  ○ Refused |
| Head of household other floor |  |
| What is the material of the head of household’s roof? | ○ Natural material  ○ Corrugated iron  ○ Roofing tiles  ○ Other  ○ Refused |
| Head of household other roof |  |
| What cooking fuel is used? | ○ Electricity  ○ Paraffin  ○ Gas  ○ Firewood  ○ Charcoal  ○ Solar  ○ Other  ○ Refused |
| Other cooking fuel |  |
| What is the water source? | ○ Piped house  ○ Piped public  ○ Public well  ○ Rain  ○ River canal  ○ Dam/pond  ○ Borehole  ○ Borehole pump  ○ Other |
| Other water source |  |
| Light source? | ○ Electricity  ○ Pressure lamp  ○ Lantern  ○ Tin lamp  ○ Fuel wood  ○ Solar  ○ Candles  ○ Kerosine  ○ Other  ○ Refused |
| Other light source |  |
| Land ownership | ○ None  ○ Own  ○ Family  ○ Rent  ○ Both  ○ Other  ○ Refused |
| Other land |  |
| Does the family keep livestock? | ○ Yes  ○ No  ○ Refused |
| Where is the livestock location? | ○ Homestead  ○ Elsewhere  ○ Both  ○ Refused |
| Which other livestock |  |
| Which livestock |  |
| Does anyone attend to livestock? | ○ Never  ○ Daily  ○ Weekly  ○ Biweekly  ○ Monthly  ○ Bimonthly  ○ Yearly  ○ N/A |
| Attend livestock frequency (second variable) |  |
| Livestock contact |  |
| Own telephone | ○ Yes  ○ No  ○ Refused |
| Own radio | ○ Yes  ○ No  ○ Refused |
| Own bicycle | ○ Yes  ○ No  ○ Refused |
| Own a motor vehicle | ○ Yes  ○ No  ○ Refused |
| Employ a domestic worker? | ○ Yes  ○ No  ○ Refused |
| What type of toilet or latrine? | ○ Flush, own  ○ Flush, shared  ○ Traditional pit latrine  ○ Ventilated improved latrine  ○ Bush/open field  ○ Other  ○ Refused |
| Other toilet latrine |  |
| Latrine location | ○ Inside your house  ○ Outside with water  ○ Outside without water  ○ No toilet  ○ Other  ○ Refused |
| Latrine location other |  |
| Latrine distance from house | ○ Less than 10m from house  ○ 10m or more from the house  ○ Other  ○ Refused |
| Other latrine distance |  |

| **Child** |  |
| --- | --- |
| What is the child’s relationship to head of household? | ○ Spouse  ○ Son  ○ Daughter  ○ Mother  ○ Father  ○ Brother  ○ Sister  ○ In-law  ○ Step son  ○ Step daughter  ○ Other  ○ Refused |
| Other child relationship to the head of household |  |
| Child’s StudyID |  |
| Child’s date of birth |  |
| Child's age (years) |  |
| Child's category | ○ Child  ○ Junior  ○ Adult |
| Is this member a child? | ○ Yes  ○ No  ○ Refused |
| Child's gender | ○ Female  ○ Male  ○ Refused |
| Child's school name |  |
| Does the child live here? | ○ Yes  ○ No  ○ Refused |
| Does the child sleep here? | ○ Yes  ○ No  ○ Refused |
| Notes |  |
