# Supplementary material for: Detection of acute dengue virus infection, with and without concurrent malaria infection, in a cohort of febrile children in Kenya, 2014–2019, by clinicians or machine learning algorithms
Source: PLOS Glob Public Health. 2023 Jul 26;3(7):e0001950. doi: 10.1371/journal.pgph.0001950 (PMC10370704; doi:10.1371/journal.pgph.0001950)
Supplement: S2 Text — (PDF) [file pgph.0001950.s008.pdf]

ID# ___________________________

tarehe:______________________
tarik:

PedsQL ™

Pediatric Quality of Life Inventory

Version 4.0 Short Form (SF15)

REPOTI YA WAZAZI KWA WATOTO WACHANGA (miaka **2-4**)
Dwoko mar jonyuol kuom nyithindo matindo(higni 2-4)

MUONGOZO
RANYISI

Katika ukurasa ufuatao kuna orodha ya mambo ambayo ni shida kwa **mtoto wako**..
Ei kwan mar otas maluwo nitie chenro mar weche ma gin chandruok/pek kuom nyathini
Tafadhali tueleze ni kiasi gani ya shida kila mmoja wapo iliyompata **mtoto wako** kwa mda wa **muezi mmoja uliopita** kwa mzunguko.
Kiyie to pimnwa ni marom nadi mar chandruok moro ka moro manoyudo nyathini kuom ndalo mar dwe achiel mokalo koluwore
:

0 kama **hakujawahi kuwa** na shida
Ka pok obet gi chandruok
1 kama **karibia hakuna** shida

2 kama **wakati mwengine** kuna shida
Ka seche moko en gi chandruok
3 kama **kila mara** kuna shida
ka ndalo duto en gi chandruok
4 kama **wakati wote** kuna shida
ka seche tee en gi chandruok

Hakuna majibu yaliyo ya sawa au yaliyo ya makosa
Kama hauelewi swali, tafadhali uliza usaidizi
Onge duoko maber kata marach ka ok iwinjo penjo to yie ikwa kony

Not to be reproduced without permission
Copyright © 1998 JW Varni, Ph.D.
All rights reserved

*Kwa **muezi mmoja** uliopita,ni kiasi gani ya **shida** mtoto wako amepata na …*
Kuom dwe achiel mosekalo, en marom nadi mar chandruok ma nyathini oseyudo gi…

**HALI YA MWILI** ***(shida na …)*** ***Chal mar del (chandruok gi..)*** Je nivigumu kwa mototo wako *Be tek nega nyathini*	**Hakuna** *Onge*	**Karibia hakuna** ***Chiegni onge***	**Wakati mwengi ne** *Seche moko*	**Kala mara** ***Ndalo duto***	**Kawati wote** *Seche te*
1. kutembea umbali wa mita kumi *wuotho bor mar mita apar*	0	1	2	3	4
2. kukimbia umbali wa mita kumi *ringo kuom bor mar mita apar*	0	1	2	3	4
3. kujihusisha na michezo au mazoezi *Timo tuke kata tugo*	0	1	2	3	4
4. kuinuwa kitu kizito *Ting'o gima pek*	0	1	2	3	4
5. kusaidia kuchua vitu vyake vya kuchezea *Konyo kuom kawo gike mag tugo*	0	1	2	3	4

**HALI ZA HISIA** ***(shida na …)*** **CHAL MAR GIK MOJAWINJO (chandruok gi…...)** Je mototo wako *Be nyathini*	**Hakuna** *onge*	**Karibia hakuna** ***Machie gni onge***	**Wakati mwengi ne** *Seche moko*	**Kala mara** ***Ndalo duto***	**Kawati wote** *Seche te*
1. huwa anahisi uoga au hofu *jawinjo ga luoro kata bwok*	0	1	2	3	4
2. anahisi huzuni *jawinjo ga lit*	0	1	2	3	4
3. anahisi hasira *jawinjo ga ich wang'*	0	1	2	3	4
4. anakuwa na wasiwasi *jabedo ga gi chuny machandre*	0	1	2	3	4

**HALI YA UHUSIANO** ***(shida na …)*** ***CHAL MAR WINJROUK (Chandruok gi……)*** Je mototo wako huwa na shida na *Be nyathini niga gi chandruok gi*	**Hakuna** *onge*	**Karibia hakuna** ***Machie gni onge***	**Wakati mwengi ne** *Seche moko*	**Kala mara** ***Ndalo duto***	**Kawati wote** *Seche te*
1. kucheza na watoto wengine *tugo gi nythindo mamoko*	0	1	2	3	4
2. watoto wengine kutotaka kucheza naye *nyithindo mamoko ok dwa tugo kode*	0	1	2	3	4
3. kuchokozwa na watoto wengine *ikwinyega gi nyithindo mamoko*	0	1	2	3	4

Not to be reproduced without permission
Copyright © 1998 JW Varni, Ph.D.
All rights reserved

tafadhali maliza sehemu hii kama mtoto wako anaenda shule
Kuom yieni duok penjo makae gi mana ka nyathini dhi skul

**HALI YA SHULE** (*shida na …*)<br>*CHAL MAR SKUL (Chandruok gi…)*<br>Je mototo wako huwa na shida na<br>*Be nyathini niga gi chandruok gi*	**Hakuna**<br>***onge***	**Karibia hakuna**<br>***Machie gni onge***	**Wakati mwengine**<br>***Seche moko***	**Kala mara**<br>***Ndalo duto***	**Kawati wote**<br>***Seche te***
1. kufanya michezo ileile ya shuleni kama wenzake<br>*Timo tuke mago mago mag skul kaka jowetege*	0	1	2	3	4
2. kukosa shule kwasababu ya kuhisi vibaya<br>*Baruok skul nikech owinjo marach*	0	1	2	3	4
3. kukosa shule ili aende kwa daktari au hospitali<br>*Baruok skul mondo odhi one laktar kata osiptal*	0	1	2	3	4

Not to be reproduced without permission
Copyright © 1998 JW Varni, Ph.D.
All rights reserved

ID# ___________________________

tarehe:_______________________
tarik

PedsQL™

Pediatric Quality of Life Inventory

Version 4.0 Short Form (SF15)

REPOTI YA MTOTO MDOGO (miaka **5-7**)
DWOKO MAR NYATHI MATIN (HIGNI 5-7)

MAAGIZO YA MWENYE KUHOJI:
RATIRO MAR JAPENJ PENJO

Nitakuuliza maswali kuhusu mambo ambayo yanaweza kuwa ni shida kwa baadhi ya watoto. Nataka kujua ni kwa kiasi gani ya shida ya mambo yoyote unayopata.
Abiro penji penjo ewi weche manyalo bedo ni gin chandruok kuom nyithindo mamoko. Adwa nge'yo ni marom nadi mag chandruok mag weche mora mora miyudo.

Onyesha mtoto nakala na aonyeshe kwa majibu kama unasoma.
Nyis nyathi kalatasni bas to otang kuom dwoko ka isomo.

Kama ni hakuna_kabisa ya shida kwako, onyesha kwa sura ya tabasamu.
Ka onge kata mar chandruok kuomi , to nyis kuom chal mar bwonjo

Kama ni wakati mwengine ya shida kwako, onyesha kwa sura ya wastani
Ka seche moko ema in gi chandruok, to nyis kuom chal ***mar kuno***

Kama shida kwako ni sana, onyesha kwa sura ya kununa
Ka chandruok kuomi en ahinya to nyis kuom chal mar kuot

Nitasoma maswali. onyesha kwa picha kunionyesha ni kwa kiasi gani ya shida uliyonayo. Tufanye majaribiyo ya kwanza.
Abiro somo penjo to siem e picha kanyisa ni kuom marom nadi mar chandruok ma in go . watem mokuongo

Not to be reproduced without permission
Copyright © 1998 JW Varni, Ph.D.
All rights reserved

	Hakuna kabisa<br><br>*Onge kata*	Wakati mwengine<br><br>*Seche moko*	Sana<br><br>Ahinya
**Je nivigumu kwako kudosa vidole vyako?**<br>Be tek ni ga ng'wonyo lith lweti?	☺	😐	☹

Uliza mtoto alize vidole vyake ili kubainisha kama swali limejibiwa sahihi. Rudia swali kama mtoto amekufanyia tofauti na matendo yake.
Penj nyathi mondo orie lith lwete mondo onyis ka penjo odwok e yo makare. nwo penjo ka nyathi otimoni mopogre.

Fikiria vile ulivyofanya kwa wiki zilizopita. Tafadhali sikiliza.
Parane kaka nitimo e wik moko mokalo. Kiyie chik iti iwinj

Baada ya kusoma kipengele, muonyeshe kwa vitendo katika nakala. Kama mtoto hajibu au anaonekana haelewi atajibu nini, soma majibu mbalimbali huku ukiotesha kwenye nyuso.
Bang' somo tang'ne ki timo e otas, ka nyathi ok dwok kata ok nenre ni owinjo ni obodwoko ango' som ne dwoko mopogre opogre ki tang'one pichni go.

**HALI YA MWILI *(shida na…)***<br>CHAL MAR DEL (chandruok gi…)<br><br>Je nivigumu kwako<br>Be tek ni ga	Hakuna kabisa<br><br>*Onge kata*	Wakati mwengine<br><br>*Seche moko*	Sana<br><br>Ahiny a
1. kutembea umbali wa kiwanja cha mpira<br>*wuotho bor mar paw mpira*	0	2	4
2. kukimbia umbali wa kiwanja cha mpira<br>*Ringo bor mar paw mpira*	0	2	4
3. kujihusisha na michezo au mazoezi<br>*timo tugo kata tuke*	0	2	4
4. kuinua kitu kizito<br>*Tingo malo gima pek*	0	2	4
5. kufanya kazi (kama kuchukua vitu vya kuchezea)<br>*Timo tich kaka kawo gigo mag tugo*	0	2	4

Kumbuka, niambie ni kwa kiasi gani shida hii umekuwa nayo katika wiki chache zilizopita.
Par mondo inyisa ni marom nadi mar shida ni misebet go kuom wige matin mosekalo

Not to be reproduced without permission
Copyright © 1998 JW Varni, Ph.D.
All rights reserved

**HALI YA HISIA *(shida na…)***<br>CHAL MAR KAKA IWINJO (Chandruok gi….)	Hakuna kabisa<br><br>*Onge kata*	Wakati mwengine<br><br>*Seche moko*	Sana<br><br>Ahiny a
1. je huwa unahisi uoga au hofu<br>*be iwinjoga luoro kata bwok*	0	2	4
2. je huwa unahisi huzuni<br>*Be iwinjo ga lit*	0	2	4
3. je huwa unahisi hasira<br>*Be iwinjo ga ich wang*	0	2	4
4. je huwa unahofu kuhusu kile kitakacho kupata<br>*Be in ga gi luoro mar gima nyalo timre ni*	0	2	4

**HALI YA UHUSUANO *(shida na …)***<br>CHAL MAR WINJRUOK (chandruok gi….)	Hakuna kabisa<br><br>*Onge kata*	Wakati mwengine<br><br>*Seche moko*	Sana<br><br>Ahiny a
1. je ni vigumu kwako kuelewana na watoto wengine<br>*be tekni ga winjruok gi nyithindo moko*	0	2	4
2. je kuna watoto wengine wanaosema hawataki kucheza na wewe<br>*be nitie nyithindo moko mawacho ni ok gidwa tugo kodi*	0	2	4
3. je watoto wengine wanakuchokoza<br>*be nyithindo moko kwinyi*	0	2	4

**HALI YA SHULE *(shida na …)***<br>CHAL MAR SKUL (Chandruok gi…)	Hakuna kabisa<br><br>*Onge kata*	Wakati mwengine<br><br>*Seche moko*	Sana<br><br>Ahiny a
1. je ni vigumu kwako kuelewa shuleni<br>*be tekni ga mako gik skul*	0	2	4
2. je huwa unasahau mambo<br>*Be wiyi wil ga gi gik moko*	0	2	4
3. je ni vigumu kwako kufanya kazi za shule<br>*Be tekni ga timo tije mag skul*	0	2	4

Not to be reproduced without permission
Copyright © 1998 JW Varni, Ph.D.
All rights reserved

Not to be reproduced without permission
Copyright © 1998 JW Varni, Ph.D.
All rights reserved

Ni kiwango gani cha shida ulichonacho?
Marom nadi mar chandruok ma in go?

Hakuna kabisa<br>*Onge kata*	Wakati mwengine<br>*seche moko*	Sana<br>*Ahinya*
☺	😐	☹

Not to be reproduced without permission

Copyright © 1998 JW Varni, PhD.
All rights reserved.

ID# ____________________________

tarehe:________________________
tarik

PedsQL™

Pediatric Quality of Life Inventory

Version 4.0 Short Form (SF15)

REPOTI YA WAZAZI KWA WATOTO WADOGO (miaka **5-7**)
DWOKO MAR JONYWOL KUOM NYITHINDO MATINDO (higni 5-7)

MUONGOZO
RATIRO

Katika ukurasa ufuatao kuna orodha ya mambo ambayo ni shida kwa **mtoto wako**..
Ei otasni nitie   chenro mar weche ma gin chandruok/pek kuom nyathini

Tafadhali tueleze ni **kiasi gani ya shida** kila mmoja wapo iliyompata **mtoto wako** kwa mda wa **muezi mmoja uliopita** kwa mzunguko.
Kiyie to pimnwa ni marom nadi mar chandruok moro ka moro manoyudo nyathini kuom ndalo mar dwe achiel mokalo koluwore
:

0 kama **hakujawahi kuwa** na shida
*Ka **pok obedo** ga gi chandruok*
1 kama **karibia hakuna** shida
*Ka **machiegni onge** chandruok*
2 kama **wakati mwengine** kuna shida
Ka **seche *moko nitie*** chandruok
3 kama **kila mara** kuna shida
*Ka **ndalo duto nitie** chandruok*
4 kama **wakati wote** kuna shida
*Ka **seche tee** nitie chandruok*

Hakuna majibu yaliyo ya sawa au yaliyo ya makosa
Kama hauelewi swali, tafadhali uliza usaidizi
Onge duoko maber kata marach ka ok iwinjo penjo to yie ikwa kony

Not to be reproduced without permission
Copyright © 1998 JW Varni, Ph.D.
All rights reserved

*Kwa **muezi mmoja** uliopita,ni kiasi gani ya **shida** mtoto wako amepata na …*
Kuom dwe achiel mosekalo, en marom nadi mar chandruok ma nyathini oseyudo…

**HALI YA MWILI** ***(shida na ...)*** *CHAL MAR DEL (chandruok gi..)* Je nivigumu kwa mototo wako *Be tekne ne ga nyathini*	**Hakuna** \ ***Onge***	**Karibia hakuna** **Machieg ni onge**	**Wakati mwengi ne** ***Seche moko***	**Kala mara** ***Ndalo duto***	**Kawati wote** ***Seche tee***
1. kutembea umbali wa kiwanja cha mpira *wuotho bor mar paw mpira*	0	1	2	3	4
2. kukimbia umbali wa kiwanja cha mpira *ringo bor mar paw mpira*	0	1	2	3	4
3. kujihusisha na michezo au mazoezi *timo tuke kata tugo*	0	1	2	3	4
4. kuinuwa kitu kizito *tingo gima pek*	0	1	2	3	4
5. kufanya kazi (kama kuchukua vitu vya kuchezea) *timo tich (kaka kawo gigene mag tugo)*	0	1	2	3	4

**HALI ZA HISIA** ***(shida na ...)*** *CHAL MAR GIK MOJAWINJO (Chandruok gi..)* Je mototo wako huwa Be nyathini	**Hakuna** ***Onge***	**Karibia hakuna** ***Machieg ni onge***	**Wakati mwengi ne** Seche moko	**Kala mara** **Ndalo duto**	**Kawati wote** ***Seche tee***
1. anahisi uoga au hofu *jawinjo ga luoro kata bwok*	0	1	2	3	4
2. anahisi huzuni *jawinjo ga lit*	0	1	2	3	4
3. anahisi hasira *jawinj ga ich wang'*	0	1	2	3	4
4. na hofu kuhusu kile kitakachompata *luoro ni gimoro biro timrene*	0	1	2	3	4

**HALI YA UHUSIANO** ***(shida na ...)*** CHAL MAR WINJRUOK (chandruok gi gi...) Je mototo wako huwa na shida na Be nyathini niga gi chandruok mar	**Hakuna** ***Onge***	**Karibia hakuna** ***Machieg ni onge***	**Wakati mwengi ne** Seche moko	**Kala mara** **Ndalo duto**	**Kawati wote** ***Seche tee***
1. kuelewana na watoto wengine *winjruok gi nyithindo mamoko*	0	1	2	3	4
2. watoto wengine kutotaka kuwa rafiki zake *Nyithindo mamoko ok dwar bedo osiepene*	0	1	2	3	4
3. kuchokozwa na watoto wengine *Ikwinye gi nyithindo mamoko*	0	1	2	3	4

Not to be reproduced without permission
Copyright © 1998 JW Varni, Ph.D.
All rights reserved

**HALI YA SHULE** ***(shida na …)***<br>CHAL MAR SKUL(Chandruok gi)<br>Je mototo wako huwa<br>*Be nyathini*	**Hakuna**<br>***Machie gni onge***	**Karibia hakuna**<br>***Machieg ni onge***	**Wakati mwengi ne**<br>**Seche moko**	**Kala mara**<br>***Sech e tee***	**Kawati wote**<br>***Seche tee***
1. anaelewa darasani<br>*winjo gik mipuonje e class*	0	1	2	3	4
2. anasahau mambo<br>*wiye wil gi gik moko*	0	1	2	3	4
3. anafanya kazi za shule<br>*be otimo tich mag skul*	0	1	2	3	4

Not to be reproduced without permission

Copyright © 1998 JW Varni, Ph.D.
All rights reserved

ID# ____________________________

Tarehe:______________________
Tarik

PedsQL ™

Pediatric Quality of Life Inventory

Version 4.0 Short Form (SF15)

REPOTI YA MTOTO (miaka **8-12**)
DWOKO MAR NYITHINDO (higni 8-12)

MUONGOZO

RATIRO

Katika ukurasa ufuatao kuna orodha ya mambo ambayo ni shida kwa **mtoto wako**..
Ei otasni moluwo nitie chenro mar weche ma gin chandruok/pek kuom nyathini
Tafadhali tueleze ni kiasi gani ya shida kila mmoja wapo iliyompata **mtoto wako** kwa mda wa **muezi mmoja uliopita** kwa mzunguko.
Kiyie to pimnwa ni marom nadi mar chandruok moro ka moro manoyudo nyathini kuom ndalo mar dwe achiel mokalo koluwore
:

0 kama **hakujawahi kuwa** na shida
Ka pok obet gi chandruok
1 kama **karibia hakuna** shida
Ka machiegni onge chandruok
2 kama **wakati mwengine** kuna shida
Ka seche moko en gi chandruok
3 kama **kila mara** kuna shida
ka ndalo duto en gi chandruok
4 kama **wakati wote** kuna shida
ka seche tee en gi chandruok

Hakuna majibu yaliyo ya sawa au yaliyo ya makosa
Kama hauelewi swali, tafadhali uliza usaidizi
Onge duoko maber kata marach ka ok iwinjo penjo to yie ikwa kony

Not to be reproduced without permission
Copyright © 1998 JW Varni, Ph.D.
All rights reserved

*Kwa **muezi mmoja** uliopita,ni kiasi gani ya **shida** imekupata …*
Kuom dwe achiel mosekalo marom nadi mar chandruok moseyudi…

**KUHUSU AFYA NA MAZOEZI *(shida na…)*** *EWI NGIMA GI TUGO(Chandruok gi)* Je nivigumu kwako Be tek niga	**Hakuna** Onge	**Karibia hakuna** **Chiegni onge**	**Wakati mwengine** Seche moko	**Kala mara** **Ndalo duto**	**Kawati wote** Seche tee
1. kutembea umbali wa kiwanja cha mpira *wuotho bor mar paw mpira*	0	1	2	3	4
2. kukimbia umbali wa kiwanja cha mpira *Ringo bor mar paw mpira*	0	1	2	3	4
3. michezo au mazoezi *tugo kata tuke*	0	1	2	3	4
4. kuinuwa kitu kizito *tingo malo gima pek*	0	1	2	3	4
5. kufanya shuhuli katika nyumba *timo tije mag ot*	0	1	2	3	4

**KUHUSU HISIA ZANGU *(shida na…)*** EWI CHAL MAR KAKA AWINJO (Chandruok gi) Je wewe huwa Be isega bedo ka	**Hakuna** Onge	**Karibia hakuna** **Chiegni onge**	**Wakati mwengine** Seche moko	**Kala mara** **Ndalo duto**	**Kawati wote** Seche tee
1. unahisi uoga au hofu *iwinjo lworo kata bwok*	0	1	2	3	4
2. una huzuni *in gi lit*	0	1	2	3	4
3. unahisi hasira *iwinjo ich wang*	0	1	2	3	4
4. unahofu kuhusu kile kitakachokupata *in gi luoro mar gima nyalo timre ni*	0	1	2	3	4

**VILE NINAVYO ELEWANA NA WENGINE *(shida na…)*** KAKA AWINJRA GI JOMOKO (Chandruok gi….) Je wewe huwa na shida na Be in ga gi chandruok gi	**Hakuna** Onge	**Karibia hakuna** **Chiegni onge**	**Wakati mwengine** Seche moko	**Kala mara** **Ndalo duto**	**Kawati wote** Seche tee
1. kuelewana na vijana wengine winjruok gi jo weteni	0	1	2	3	4
2. vijana wengine kutotaki kuwa rafiki zako joweteni moko ok dwa bedo osiepeni	0	1	2	3	4
3. vijana wengine wanaokuchokoza joweteni moko kwinyiga	0	1	2	3	4

Not to be reproduced without permission

Copyright © 1998 JW Varni, Ph.D.
All rights reserved

**KUHUSU SHULE** ***(shida na…)***<br>EWI SKUL (Chandruok gi…)<br><br>Je wewe huwa na shida na<br>Be in ga gi chandruok gi	**Hakuna**<br><br>Onge	**Karibia hakuna**<br><br>**Chiegni onge**	**Wakati mwengi ne**<br><br>Seche moko	**Kala mara**<br><br>**Ndalo duto**	**Kawati wote**<br><br>Seche tee
1. kuelewa darasani<br>*mako gik mopuonj e klas*	0	1	2	3	4
2. sahau mambo<br>*wich wil gi gik moko*	0	1	2	3	4
3 kufanya kazi za shule<br>*Timo tije mag skul*	0	1	2	3	4

Not to be reproduced without permission
Copyright © 1998 JW Varni, Ph.D.
All rights reserved

ID# ____________________________

Tarehe:________________________

PedsQL ™

Pediatric Quality of Life Inventory

Version 4.0 Short Form (SF15)

REPOTI YA WAZAZI KWA WATOTO (miaka **8-12**)
DWOKO MAR JONYUOL KUOM NYITHINDO (Higni 8-12)

MUONGOZO
RATIRO

Katika ukurasa ufuatao kuna orodha ya mambo ambayo ni shida kwa **mtoto wako**..
Ei otasni nitie chenro mar weche ma gin chandruok/pek kuom nyathini

Tafadhali tueleze ni **kiasi gani ya shida** kila mmoja wapo iliyompata **mtoto wako** kwa mda wa **muezi mmoja uliopita** kwa mzunguko.
Kiyie to pimnwa ni marom nadi mar chandruok moro ka moro manoyudo nyathini kuom ndalo mar dwe achiel mokalo koluwore
:

0 kama **hakujawahi kuwa** na shida
*Ka **pok obedo** ga gi chandruok*
1 kama **karibia hakuna** shida
*Ka **chiegni onge** chandruok*
2 kama **wakati mwengine** kuna shida
Ka **seche *moko nitie*** chandruok
3 kama **kila mara** kuna shida
*Ka **ndalo duto nitie** chandruok*
4 kama **wakati wote** kuna shida
*Ka **seche tee** nitie chandruok*

Hakuna majibu yaliyo ya sawa au yaliyo ya makosa
Kama hauelewi swali, tafadhali uliza usaidizi
Onge duoko maber kata marach ka ok iwinjo penjo to yie ikwa kony

Not to be reproduced without permission
Copyright © 1998 JW Varni, Ph.D.
All rights reserved

*Kwa **muezi mmoja** uliopita,ni kiasi gani ya **shida** mtoto wako amepata na …*
Kuom dwe achiel mokalo en marom nadi mar chandruok ma nyathini ose yudo gi….

**HALI YA MWILI** ***(shida na ...)***<br>*CHAL MAR DEL (Chandruok gi,,,,)*<br>Je nivigumu kwa mtoto wako<br>*Be tek ne ga nyathini*	**Hakuna**<br>onge	**Karibia hakuna**<br>**Machieg ni onge**	**Wakati mwengi ne**<br>Seche moko	**Kala mara**<br>**Ndalo duto**	**Kawati wote**<br>Seche tee
1. kutembea umbali wa kiwanja cha mpira<br>*Wotho bor mar paw mpira*	0	1	2	3	4
2. kukimbia umbali wa kiwanja cha mpira<br>*ringo bor mar paw mpira*	0	1	2	3	4
3. kujihusisha na michezo au mazoezi<br>*Donjo e tugo kata tuke*	0	1	2	3	4
4. kuinuwa kitu kizito<br>*tingo malo gima pek*	0	1	2	3	4
5. kufanya kazi ndani ya nyumba<br>*timo tich ei oot*	0	1	2	3	4

**HALI ZA HISIA** ***(shida na ...)***<br>*CHAL MAR KAKA OWINJO (Shida gi..)*<br>Je mtoto wako huwa<br>*Be nyathini*	**Hakuna**<br>onge	**Karibia hakuna**<br>**Machieg ni onge**	**Wakati mwengi ne**<br>Seche moko	**Kala mara**<br>**Ndalo duto**	**Kawati wote**<br>Seche tee
1. anahisi uoga au hofu<br>*owinjo luoro kata bwok*	0	1	2	3	4
2. anahisi huzuni<br>*owinjo lit*	0	1	2	3	4
3. anahisi hasira<br>*owinjo ich wang*	0	1	2	3	4
4. na hofu kuhusu kile kitakachompata<br>*gi bwok kuom gima nyalo timorene*	0	1	2	3	4

**HALI YA UHUSIANO** ***(shida na ...)***<br>YOR WINJRUOK (Chandruok gi….)<br>Je mtoto wako huwa na shida na<br>*Be nyathini niga gi chandruok gi*	**Hakuna**<br>onge	**Karibia hakuna**<br>**Machieg ni onge**	**Wakati mwengi ne**<br>Seche moko	**Kala mara**<br>**Ndalo duto**	**Kawati wote**<br>Seche tee
1. kuelewana na watoto wengine<br>*winjruok gi nyithindo moko*	0	1	2	3	4
2. watoto wengine kutotaka kuwa rafiki yake<br>*Nyithindo mok ok dwa bedo osiepege*	0	1	2	3	4
3. kuchokozwa na watoto wengine<br>*Ikwinye gi nyithindo moko*	0	1	2	3	4

Not to be reproduced without permission
Copyright © 1998 JW Varni, PhD.
All rights reserved

**HALI YA SHULE** ***(shida na ...)***<br>CHAL MAR SKUL(chandruok gi…)<br>Je mtoto wako huwa na shida na<br>*Be nyathini nig a gi chandruok gi…*	**Hakuna**<br><br>onge	**Karibia hakuna**<br><br>**Machieg ni onge**	**Wakati mwengine**<br>Seche moko	**Kala mara**<br><br>**Ndalo duto**	**Kawati wote**<br>Seche tee
1. kuaelewa darasani<br>*mako gig moko e klas*	0	1	2	3	4
2. kusahau mambo<br>*wiye wil gi gik moko*	0	1	2	3	4
4. kufanya kazi za shule<br>*timo tije ge mag skul*	0	1	2	3	4

Not to be reproduced without permission
Copyright © 1998 JW Varni, PhD. All rights reserved

ID# ____________________________

Tarehe:______________________
tarik

PedsQL ™

Pediatric Quality of Life Inventory

Version 4.0 Short Form (SF15)

RIPOTI YA VIJANA (miaka **13-18**)
DWOKO MAG RAWERA (Higni 13-18)

MUONGOZO
RATIRO

Katika ukurasa ufuatao kuna orodha ya vitu ambavyo ni shida kwako..
Ei otasni moluwore nitie   chenro mar weche ma gin chandruok/pek kuomi

Tafadhali tueleze ni kwa **kiasi gani ya kila shida** ya kila mmoja wapo uliyo nayo kwa mda wa **muezi mmoja uliopita** kwa mzunguko.
Kiyie to pimnwa ni marom nadi mar chandruok moro ka moro mise bet go kwom ndalo mar dwe achiel mosekalo maluwore
:

0 kama **hakujawahi kuwa** na shida
*Ka **pok ibedo** ga gi chandruok*
1 kama **karibia hakuna** shida
*Ka ma**chiegni onge** chandruok*
2 kama **wakati mwengine** kuna shida
Ka **seche *moko nitie*** chandruok
3 kama **kila mara** kuna shida
*Ka **ndalo duto nitie** chandruok*
4 kama **wakati wote** kuna shida
*Ka **seche tee** nitie chandruok*

Hakuna majibu yaliyo ya sawa au yaliyo ya makosa
Kama hauelewi swali, tafadhali uliza usaidizi
Onge duoko maber kata marach ka ok iwinjo penjo to yie ikwa kony

Not to be reproduced without permission
Copyright © 1998 JW Varni, Ph.D.
All rights reserved

*Kwa **muezi mmoja** uliopita,ni kiasi gani ya **shida** imekupata …*
Kuom dwe achiel mosekalo marom nadi mar chandruok moseyudi…

**KUHUSU AFYA NA MAZOEZI** ***(shida na…)***<br>***EWI NGIMA GI TUGO(Chandruok gi)***<br><br>Je nivigumu kwako<br>Be tek ni ga	**Hakuna**<br>Onge	**Karibia hakuna**<br>**Chiegni onge**	**Wakati mwengine**<br>Seche moko	**Kala mara**<br>**Ndalo duto**	**Kawati wote**<br>Seche tee
1. kutembea umbali wa kiwanja cha mpira<br>*wuotho bor mar paw mpira*	0	1	2	3	4
2. kukimbia umbali wa kiwanja cha mpira<br>*Ringo bor mar paw mpira*	0	1	2	3	4
3. kufanya michezo au mazoezi<br>*tugo kata tuke*	0	1	2	3	4
4. kuinuwa kitu kizito<br>*tingo malo gima pek*	0	1	2	3	4
5. kufanya kazi ndani ya nyumba<br>*timo tije mag ot*	0	1	2	3	4

**KUHUSU HISIA ZANGU** ***(shida na…)***<br>EWI CHAL MAR KAKA AWINJO (Chandruok gi)<br><br>Je wewe huwa na<br>Be isega bedo ka	**Hakuna**<br>Onge	**Karibia hakuna**<br>**Chiegni onge**	**Wakati mwengine**<br>Seche moko	**Kala mara**<br>**Ndalo duto**	**Kawati wote**<br>Seche tee
1. unahisi uoga au hofu<br>*iwinjo lworo kata bwok*	0	1	2	3	4
2. una huzuni<br>*in gi lit*	0	1	2	3	4
3. unahisi hasira<br>*iwinjo ich wang*	0	1	2	3	4
4. unahofu kuhusu kile kitakachokupata<br>*in gi luoro mar gima nyalo timre ni*	0	1	2	3	4

**VILE NINAVYO ELEWANA NA WENGINE** ***(shida na…)***<br>KAKA AWINJRA GI JOMOKO (Chandruok gi….)<br><br>Je wewe huwa na shida na<br>Be in ga gi chandruok gi	**Hakuna**<br>Onge	**Karibia hakuna**<br>**Chiegni onge**	**Wakati mwengine**<br>Seche moko	**Kala mara**<br>**Ndalo duto**	**Kawati wote**<br>Seche tee
1. kuelewana na vijana wengine<br>winjruok gi jo weteni	0	1	2	3	4
2. vijana wengine hawataki kuwa rafiki zangu<br>jowetega ok dwa bedo osiepena	0	1	2	3	4
3. vijana wengine wananichokoza<br>jowetega moko kwinya	0	1	2	3	4

Not to be reproduced without permission
Copyright © 1998 JW Varni, Ph.D.
All rights reserved

**KUHUSU SHULE *(shida na…)*** EWI SKUL (Chandruok gi…)<br><br>Je wewe huwa na shida na<br>Be in ga gi chandruok gi	**Hakuna**<br><br>Onge	**Karibia hakuna**<br><br>**Chiegni onge**	**Wakati mwengi ne**<br><br>Seche moko	**Kala mara**<br><br>**Ndalo duto**	**Kawati wote**<br><br>Seche tee
1. kuelewa darasani<br>*mako gik mopuonj e klas*	0	1	2	3	4
2. kusahau mambo<br>*wich wil gi gik moko*	0	1	2	3	4
3 kufanya kazi za shule<br>*Timo tije mag skul*	0	1	2	3	4

Not to be reproduced without permission

Copyright © 1998 JW Varni, Ph.D.
All rights reserved

ID#________________________

Tarehe:______________________
tarik

PedsQL ™

Pediatric Quality of Life Inventory

Version 4.0 Short Form (SF15)

REPOTI YA WAZAZI KWA VIJANA (miaka **13-18**)
DWOKO MAR JONYUOL KUOM RAWERA (higni 13-18)

MUONGOZO
RATIRO

Katika ukurasa ufuatao kuna orodha ya mambo ambayo ni shida kwa **mtoto wako**..
Ei otasni nitie chenro mar weche ma gin chandruok/pek kuom nyathini

Tafadhali tueleze ni **kiasi gani ya shida** kila mmoja wapo iliyompata **mtoto wako** kwa mda wa **muezi mmoja uliopita** kwa mzunguko.
Kiyie to pimnwa ni marom nadi mar chandruok moro ka moro manoyudo nyathini kuom ndalo mar dwe achiel mokalo koluwore
:

0 kama **hakujawahi kuwa** na shida
*Ka **pok obedo** ga gi chandruok*
1 kama **karibia hakuna** shida
*Ka **chiegni onge** chandruok*
2 kama **wakati mwengine** kuna shida
Ka **seche *moko nitie*** chandruok
3 kama **kila mara** kuna shida
*Ka **ndalo duto nitie** chandruok*
4 kama **wakati wote** kuna shida
*Ka **seche tee** nitie chandruok*

Hakuna majibu yaliyo ya sawa au yaliyo ya makosa
Kama hauelewi swali, tafadhali uliza usaidizi
Onge duoko maber kata marach ka ok iwinjo penjo to yie ikwa kony

 Not to be reproduced without permission Copyright © 1998 JW Varni, Ph.D. 03/00
All rights reserved

Kwa muezi mmoja uliopita,ni kiasi gani ya shida kijana wako amepata na …
Kuom dwe achiel mokalo en marom nadi mar chandruok ma nyathini ose yudo gi….

**HALI YA MWILI** ***(shida na…)*** CHAL MAR DEL (Chandruok gi,,,,) Je nivigumu kwa mtoto wako *Be tek ne ga nyathini*	**Hakuna** onge	**Karibia hakuna** **Machie gni onge**	**Wakati mwengine** Seche moko	**Kala mara** **Ndalo duto**	**Kawati wote** Seche tee
1. kutembea umbali wa kiwanja cha mpira *Wotho bor mar paw mpira*	0	1	2	3	4
2. kukimbia umbali wa kiwanja cha mpira *ringo bor mar paw mpira*	0	1	2	3	4
3. kufanya michezo au mazoezi *Donjo e tugo kata tuke*	0	1	2	3	4
4. kuinuwa kitu kizito *tingo malo gima pek*	0	1	2	3	4
5. kufanya kazi ndani ya nyumba *timo tich ei oot*	0	1	2	3	4

**HALI ZA HISIA** ***(shida na …)*** CHAL MAR KAKA OWINJO (Shida gi..) Je mtoto wako huwa *Be nyathini*	**Hakuna** onge	**Karibia hakuna** **Machie gni onge**	**Wakati mwengine** Seche moko	**Kala mara** **Ndalo duto**	**Kawati wote** Seche tee
1. anahisi uoga au hofu 2. *owinjo luoro kata bwok*	0	1	2	3	4
3. anahisi huzuni *owinjo lit*	0	1	2	3	4
4. anahisi hasira *owinjo ich wang*	0	1	2	3	4
5. anahofu kuhusu kile kitakachompata *gi bwok kuom gima nyalo timorene*	0	1	2	3	4

**HALI YA UHUSIANO** ***(shida na …)*** YOR WINJRUOK (Chandruok gi….) Je mtoto wako huwa na shida na *Be nyathini niga gi chandruok gi*	**Hakuna** onge	**Karibia hakuna** **Machie gni onge**	**Wakati mwengine** Seche moko	**Kala mara** **Ndalo duto**	**Kawati wote** Seche tee
1. kuelewana na vijana wengine *winjruok gi nyithindo moko*	0	1	2	3	4
2. vijana wengine kutotaka kuwa rafiki yake *Nyithindo mok ok dwa bedo osiepege*	0	1	2	3	4
3. kuchokozwa na vijana wengine *Ikwinye gi nyithindo moko*	0	1	2	3	4

Not to be reproduced without permission
Copyright © 1998 JW Varni, Ph.D.
All rights reserved

**HALI YA SHULE** ***(shida na …)***<br>CHAL MAR SKUL(chandruok gi…)<br>Je mtoto wako huwa na shida na<br>*Be nyathini nig a gi chandruok gi…*	**Hakun a**<br><br>onge	**Karibia hakuna**<br><br>**Machie gni onge**	**Wakati mweng ine**<br>Seche moko	**Kala mara**<br><br>**Ndalo duto**	**Kawati wote**<br>Seche tee
1. kuelewa darasani<br>*mako gig moko e klas*	0	1	2	3	4
2. kusahau mambo<br>*wiye wil gi gik moko*	0	1	2	3	4
3 kufanya kazi za shule<br>*timo tije ge mag skul*	0	1	2	3	4

Not to be reproduced without permission

Copyright © 1998 JW Varni, Ph.D.
All rights reserved

ID# ___________________________

Tarik: ________________________

PedsQL ™

Pediatric Quality of Life Inventory

Version 4.0 Short Form (SF15)

DWOKO MAR JONYUOL KUOM NYITHINDO MATINDO (higni 2-4)

RANYISI

Ei kwan mar otas maluwo nitie chenro mar weche ma gin chandruok/pek **kuom nyathini**

Kiyie to pimnwa ni marom nadi mar chandruok moro ka moro manoyudo **nyathini kuom** ndalo mar **dwe achiel mokalo** koluwore

:

0 Ka **pok obet gi** chandruok
1 Ka **machiegni onge** chandruok
2 Ka **seche moko** en gi chandruok
3 Ka **ndalo duto** en gi chandruok
4 Ka **seche tee** en gi chandruok

Onge duoko maber kata marach ka ok iwinjo penjo to yie ikwa kony

Not to be reproduced without permission

Copyright © 1998 JW Varni, Ph.D.
All rights reserved

*Kuom **dwe achiel** mosekalo, en marom nadi mar **chandruok** ma nyathini oseyudo gi…*

**CHAL MAR DEL (chandruok gi..)**<br><br>Be tek nega nyathini	***Onge***	***Chiegni onge***	***Seche moko***	***Ndalo duto***	***Seche te***
1.Wuotho bor mar mita apar	0	1	2	3	4
2. Ringo kuom bor mar mita apar	0	1	2	3	4
3. Timo tuke kata tugo	0	1	2	3	4
4. Ting'o gima pek	0	1	2	3	4
5. Konyo kuom kawo gike mag tugo	0	1	2	3	4

**CHAL MAR GIK MOJAWINJO (chandruok gi…...)**<br><br>Be nyathini	**Onge**	**Machie gni onge**	**Seche moko**	**Ndalo duto**	**Seche te**
1. Jawinjo ga luoro kata bwok	0	1	2	3	4
2. Jawinjo ga lit	0	1	2	3	4
3. Jawinjo ga ich wang'	0	1	2	3	4
4. Jabedo ga gi chuny machandre	0	1	2	3	4

**CHAL MAR WINJROUK (CHANDRUOK GI…...)**<br><br>Be nyathini niga gi chandruok gi	**Onge**	**Machie gni onge**	**Seche moko**	**Ndalo duto**	**Seche te**
1. Tugo gi nythindo mamoko	0	1	2	3	4
2. Nyithindo mamoko ok dwa tugo kode	0	1	2	3	4
3. Ikwinyega gi nyithindo mamoko	0	1	2	3	4

***Kuom yieni duok penjo makae gi mana ka nyathini dhi skul**

CHAL MAR SKUL (CHANDRUOK GI…)<br><br>Be nyathini niga gi chandruok gi	**Onge**	**Machie gni onge**	**Seche moko**	**Ndalo duto**	**Seche te**
1. Timo tuke mago mago mag skul kaka jowetege	0	1	2	3	4
2. Baruok skul nikech owinjo marach	0	1	2	3	4
3. Baruok skul mondo odhi one laktar kata osiptal	0	1	2	3	4

Not to be reproduced without permission
Copyright © 1998 JW Varni, Ph.D.
All rights reserved

ID# ___________________________
Tarik: _________________________

PedsQL™

Pediatric Quality of Life Inventory

Version 4.0 Short Form (SF15)

DWOKO MAR NYATHI MATIN (higni 5-7)

RATIRO MAR JAPENJ PENJO:

Abiro penji penjo ewi weche manyalo bedo ni gin chandruok kuom nyithindo mamoko. Adwa nge'yo ni marom nadi mag chandruok mag weche mora mora miyudo.

Nyis nyathi kalatasni bas to otang kuom dwoko ka isomo.

Ka onge kata matin mar chandruok kuomi , to nyis kuom chal mar bwonjo.

Ka seche moko ema in gi chandruok, to nyis kuom chal mar kuno.

Ka chandruok kuomi en ahinya to nyis kuom chal mar kuot.

Abiro somo penjo to siem e picha kanyisa ni kuom marom nadi mar chandruok ma in go . watem mokuongo

	Onge kata	*Seche moko*	Ahinya
**Be tek ni ga ng'wonyo lith lweti?**	☺	😐	☹

Penj nyathi mondo orie lith lwete mondo onyis ka penjo odwok e yo makare. nwo penjo ka nyathi otimoni mopogre.

Not to be reproduced without permission
Copyright © 1998 JW Varni, Ph.D.
All rights reserved

Parane kaka nitimo e wik moko mokalo. Ikwayi ni ichik iti iwinj.

Bang' somo, tang'ne kuom tim e otas, ka nyathi ok dwok kata ok nenre ni owinjo ni obodwoko ango' som ne dwoko mopogre opogre ki tang'one pichni go.

**CHAL MAR DEL (CHANDRUOK GI…)**<br><br>Be tek ni ga	***Onge kata***	***Seche moko***	**Ahinya**
1. Wuotho bor mar paw mpira	0	2	4
2. Ringo bor mar paw mpira	0	2	4
3. Timo tugo kata tuke	0	2	4
4. Tingo malo gima pek	0	2	4
5. Timo tich kaka kawo gigo mag tugo	0	2	4

Par mondo inyisa ni marom nadi mar shida ni misebet go kuom wige matin mosekalo.

**CHAL MAR KAKA IWINJO (Chandruok gi….)**	***Onge kata***	***Seche moko***	**Ahinya**
1. Be iwinjoga luoro kata bwok	0	2	4
2. Be iwinjo ga lit	0	2	4
3. Be iwinjo ga ich wang	0	2	4
4. Be in ga gi luoro mar gima nyalo timre ni	0	2	4

**CHAL MAR WINJRUOK (CHANDRUOK GI….)**	***Onge kata***	***Seche moko***	**Ahinya**
1. Be tekni ga winjruok gi nyithindo moko	0	2	4
2. Be nitie nyithindo moko mawacho ni ok gidwa tugo kodi	0	2	4
3. Be nyithindo moko kwinyi ga	0	2	4

**CHAL MAR SKUL (CHANDRUOK GI…)**	***Onge kata***	***Seche moko***	**Ahinya**
1. Be tekni ga mako gik skul	0	2	4
2. Be wiyi wil ga gi gik moko	0	2	4
3. Be tekni ga timo tije mag skul	0	2	4

Not to be reproduced without permission
Copyright © 1998 JW Varni, Ph.D.
All rights reserved

Marom nadi mar chandruok ma in go?

Onge kata	*Seche moko*	*Ahinya*
☺	😐	☹

Not to be reproduced without permission
Copyright © 1998 JW Varni, PhD.
All rights reserved.

ID# ___________________________

Tarik:______________________

PedsQL ™

Pediatric Quality of Life Inventory

Version 4.0 Short Form (SF15)

DWOKO MAR JONYUOL KUOM NYITHINDO MATINDO (higni 5-7)

RATIRO

Ei otasni nitie chenro mar weche ma gin chandruok/pek kuom **nyathini**

Kiyie to pimnwa ni **marom nadi** mar chandruok moro ka moro manoyudo **nyathini** kuom ndalo mar **dwe achiel mokal**o koluwore
:

0 Ka **pok obedo** ga gi chandruok
1 Ka **machiegni onge** chandruok
2 Ka **seche moko nitie** chandruok
3 Ka **ndalo duto nitie** chandruok
4 Ka **seche tee** nitie chandruok

Onge duoko maber kata marach
Ka ok iwinjo penjo to yie ikwa kony

Not to be reproduced without permission
Copyright © 1998 JW Varni, Ph.D.
All rights reserved

*Kuom **dwe achiel** mosekalo, en marom nadi mar **chandruok** ma nyathini oseyudo…*

**CHAL MAR DEL** (**CHANDRUOK GI..**)<br>Be tekne ne ga nyathini	**Onge**	**Machieg ni onge**	**Seche moko**	**Ndalo duto**	**Seche tee**
1. Wuotho bor mar paw mpira	0	1	2	3	4
2. Ringo bor mar paw mpira	0	1	2	3	4
3. Timo tuke kata tugo	0	1	2	3	4
4. Ting'o gima pek	0	1	2	3	4
5. Timo tich (kaka kawo gigene mag tugo)	0	1	2	3	4

**CHAL MAR GIK MOJAWINJO** (**CHANDRUOK GI..**)<br>Be nyathini	**Onge**	**Machieg ni onge**	**Seche moko**	**Ndalo duto**	**Seche tee**
1. Jawinjo ga luoro kata bwok	0	1	2	3	4
2. Jawinjo ga lit	0	1	2	3	4
3. Jawinj ga ich wang'	0	1	2	3	4
4. Luoro ni gimoro biro timrene	0	1	2	3	4

**CHAL MAR WINJRUOK** (**CHANDRUOK GI GI...**)<br>Be nyathini niga gi chandruok mar	**Onge**	**Machieg ni onge**	**Seche moko**	**Ndalo duto**	**Seche tee**
1. Winjruok gi nyithindo mamoko	0	1	2	3	4
2. Nyithindo mamoko ok dwar bedo osiepene	0	1	2	3	4
3. Ikwinye gi nyithindo mamoko	0	1	2	3	4

CHAL MAR SKUL (CHANDRUOK GI)<br>Be nyathini	**Onge**	**Machieg ni onge**	**Seche moko**	**Seche tee**	**Seche tee**
1. Winjoga gik mipuonje e class	0	1	2	3	4
2. Wiye wil gi gik moko	0	1	2	3	4
3. Be otimo tich mag skul	0	1	2	3	4

Not to be reproduced without permission

Copyright © 1998 JW Varni, Ph.D.
All rights reserved

ID# ____________________________

Tarik:________________________

PedsQL ™

Pediatric Quality of Life Inventory

Version 4.0 Short Form (SF15)

DWOKO MAR NYITHINDO (higni 8-12)

RATIRO

Ei otasni moluwo nitie chenro mar weche ma gin chandruok/pek kuom **nyathini**

Kiyie to pimnwa ni marom nadi mar chandruok moro ka moro manoyudo **nyathini** kuom ndalo mar **dwe achiel mokalo** koluwore

:

0 Ka **pok** obet gi chandruok
1 Ka **machiegni** onge chandruok
2 Ka **seche moko** en gi chandruok
3 ka **ndalo duto** en gi chandruok
4 ka **seche tee** en gi chandruok

Onge duoko maber kata marach
Ka ok iwinjo penjo to yie ikwa kony

Not to be reproduced without permission

Copyright © 1998 JW Varni, Ph.D.
All rights reserved

*Kuom **dwe achiel** mosekalo marom nadi mar **chandruok** moseyudi…*

**EWI NGIMA GI TUGO (Chandruok gi…)**<br><br>Be tek niga	**Onge**	**Chiegni onge**	**Seche moko**	**Ndalo duto**	**Seche tee**
1. Wuotho bor mar paw mpira	0	1	2	3	4
2. Ringo bor mar paw mpira	0	1	2	3	4
3. Tugo kata tuke	0	1	2	3	4
4. Tingo malo gima pek	0	1	2	3	4
5. Timo tije mag ot	0	1	2	3	4

**EWI CHAL MAR KAKA AWINJO (Chandruok gi…)**<br><br>Be isega bedo ka	**Onge**	**Chiegni onge**	**Seche moko**	**Ndalo duto**	**Seche tee**
1. Iwinjo lworo kata bwok	0	1	2	3	4
2. In gi lit	0	1	2	3	4
3. Iwinjo ich wang	0	1	2	3	4
4. In gi luoro mar gima nyalo timre ni	0	1	2	3	4

**KAKA AWINJRA GI JOMOKO (CHANDRUOK GI….)**<br><br>Be in ga gi chandruok gi	**Onge**	**Chiegni onge**	**Seche moko**	**Ndalo duto**	**Seche tee**
1. Winjruok gi jo weteni	0	1	2	3	4
2. Joweteni moko ok dwa bedo osiepeni	0	1	2	3	4
3. Joweteni moko kwinyiga	0	1	2	3	4

**EWI SKUL (CHANDRUOK GI…)**<br><br>Be in ga gi chandruok gi	**Onge**	**Chiegni onge**	**Seche moko**	**Ndalo duto**	**Seche tee**
1. Mako gik mopuonj e klas	0	1	2	3	4
2. Wich wil gi gik moko	0	1	2	3	4
3. Tmo tije mag skul	0	1	2	3	4

Not to be reproduced without permission
Copyright © 1998 JW Varni, Ph.D.
All rights reserved

ID# ___________________________

Tarik:_______________________

PedsQL ™

Pediatric Quality of Life Inventory

Version 4.0 Short Form (SF15)

DWOKO MAR JONYUOL KUOM NYITHINDO (higni 8-12)

RATIRO

Ei otasni nitie chenro mar weche ma gin chandruok/pek **kuom nyathini**

Kiyie to pimnwa ni **marom nadi mar chandruok** moro ka moro manoyudo **nyathini kuom** ndalo mar dwe achiel mokalo koluwore
:

0 Ka **pok obedo** ga gi chandruok
1 Ka **chiegni onge** chandruok
2 Ka **seche moko nitie** chandruok
3 Ka **ndalo duto nitie** chandruok
4 Ka **seche tee** nitie chandruok

Onge duoko maber kata marach
Ka ok iwinjo penjo to yie ikwa kony

Not to be reproduced without permission
Copyright © 1998 JW Varni, Ph.D.
All rights reserved

*Kuom **dwe achiel** mokalo en marom nadi mar chandruok ma nyathini ose yudo gi….*

**CHAL MAR DEL (CHANDRUOK GI,,,,)**<br>*Be tek ne ga nyathini*	**Onge**	**Machieg ni onge**	**Seche moko**	**Ndalo duto**	**Seche tee**
1. Wotho bor mar paw mpira	0	1	2	3	4
2. Ringo bor mar paw mpira	0	1	2	3	4
3. Donjo e tugo kata tuke	0	1	2	3	4
4. Tingo malo gima pek	0	1	2	3	4
5. Timo tich ei oot	0	1	2	3	4

**CHAL MAR KAKA OWINJO (SHIDA GI..)**<br>Be nyathini	**Onge**	**Machieg ni onge**	**Seche moko**	**Ndalo duto**	**Seche tee**
1. Owinjo luoro kata bwok	0	1	2	3	4
2. Owinjo lit	0	1	2	3	4
3. Owinjo ich wang	0	1	2	3	4
4. Ni ga gi bwok kuom gima nyalo timorene	0	1	2	3	4

**YOR WINJRUOK (CHANDRUOK GI….)**<br>*Be nyathini niga gi chandruok gi*	**Onge**	**Machieg ni onge**	**Seche moko**	**Ndalo duto**	**Seche tee**
1. Winjruok gi nyithindo moko	0	1	2	3	4
2. Nyithindo mok ok dwa bedo osiepege	0	1	2	3	4
3. Ikwinye gi nyithindo moko	0	1	2	3	4

**CHAL MAR SKUL (CHANDRUOK GI…)**<br>Be nyathini nig a gi chandruok gi…	**Onge**	**Machieg ni onge**	**Seche moko**	**Ndalo duto**	**Seche tee**
1. Mako gig moko e klas	0	1	2	3	4
2. Wiye wil gi gik moko	0	1	2	3	4
3. Timo tije ge mag skul	0	1	2	3	4

Not to be reproduced without permission
Copyright © 1998 JW Varni, PhD.
All rights reserved

ID# ______________________________

TarIK:__________________________

PedsQL ™

Pediatric Quality of Life Inventory

Version 4.0 Short Form (SF15)

DWOKO MAG RAWERA (Higni 13-18)

RATIRO

Ei otasni moluwore nitie chenro mar weche ma gin chandruok/pek kuomi

Kiyie to pimnwa ni **marom nadi mar chandruok** moro ka moro mise bet go kwom ndalo mar **dwe achiel mosekalo** maluwore
:

0 Ka **pok ibedo** ga gi chandruok
1 Ka ma**chiegni onge** chandruok
2 Ka **seche moko nitie** chandruok
3 Ka **ndalo duto nitie** chandruok
4 Ka **seche tee** nitie chandruok

Onge duoko maber kata marach
ka ok iwinjo penjo to yie ikwa kony

Not to be reproduced without permission

Copyright © 1998 JW Varni, Ph.D.
All rights reserved

*Kuom **dwe achiel** mosekalo marom nadi mar **chandruok** moseyudi…*

**EWI NGIMA GI TUGO(CHANDRUOK GI)**<br>Be tek ni ga	**Onge**	**Chiegni onge**	**Seche moko**	**Ndalo duto**	**Seche tee**
1. Wuotho bor mar paw mpira	0	1	2	3	4
2. Ringo bor mar paw mpira	0	1	2	3	4
3. Tugo kata tuke	0	1	2	3	4
4. Tingo malo gima pek	0	1	2	3	4
5. Timo tije mag ot	0	1	2	3	4

**EWI CHAL MAR KAKA AWINJO (CHANDRUOK GI)**<br>Be isega bedo ka	**Onge**	**Chiegni onge**	**Seche moko**	**Ndalo duto**	**Seche tee**
1. Iwinjo lworo kata bwok	0	1	2	3	4
2. In gi lit	0	1	2	3	4
3. Iwinjo ich wang	0	1	2	3	4
4. In gi luoro mar gima nyalo timre ni	0	1	2	3	4

**KAKA AWINJRA GI JOMOKO (CHANDRUOK GI….)**<br>Be in ga gi chandruok gi	**Onge**	**Chiegni onge**	**Seche moko**	**Ndalo duto**	**Seche tee**
1. Winjruok gi jo weteni	0	1	2	3	4
2. Jowetega ok dwa bedo osiepena	0	1	2	3	4
3. Jowetega moko kwinya	0	1	2	3	4

**EWI SKUL (CHANDRUOK GI…)**<br>Be in ga gi chandruok gi	**Onge**	**Chiegni onge**	**Seche moko**	**Ndalo duto**	**Seche tee**
1. Mako gik mopuonj e klas	0	1	2	3	4
2. Wich wil gi gik moko	0	1	2	3	4
3. Timo tije mag skul	0	1	2	3	4

Not to be reproduced without permission
Copyright © 1998 JW Varni, Ph.D.
All rights reserved

ID#___________________________

Tarik:________________________

PedsQL ™

Pediatric Quality of Life Inventory

Version 4.0 Short Form (SF15)

DWOKO MAR JONYUOL KUOM RAWERA (higni 13-18)

RATIRO..

Ei otasni nitie chenro mar weche ma gin chandruok/pek kuom **nyathini**

Kiyie to pimnwa ni **marom nadi mar** chandruok moro ka moro manoyudo nyathini kuom ndalo mar **dwe achiel mokalo** koluwore

:

0 Ka **pok obedo** ga gi chandruok
1 Ka **chiegni onge** chandruok
2 Ka **seche moko nitie** chandruok
3 Ka **ndalo duto nitie** chandruok
4 Ka **seche tee** nitie chandruok

Onge duoko maber kata marach
Ka ok iwinjo penjo to yie ikwa kony

Not to be reproduced without permission
Copyright © 1998 JW Varni, Ph.D. 03/00
All rights reserved

Kuom dwe achiel mokalo en marom nadi mar chandruok ma nyathini ose yudo gi….

CHAL MAR DEL (CHANDRUOK GI….)<br>Be tek ne ga nyathini	Onge	Machie gni onge	Seche moko	Ndalo duto	Seche tee
1. Wotho bor mar paw mpira	0	1	2	3	4
2. Ringo bor mar paw mpira	0	1	2	3	4
3. Donjo e tugo kata tuke	0	1	2	3	4
4. Ting'o malo gima pek	0	1	2	3	4
5. Timo tich ei oot	0	1	2	3	4

CHAL MAR KAKA OWINJO (SHIDA GI….)<br>Be nyathini	Onge	Machie gni onge	Seche moko	Ndalo duto	Seche tee
1. Owinjo luoro kata bwok	0	1	2	3	4
2. Owinjo lit	0	1	2	3	4
4. Owinjo ich wang	0	1	2	3	4
3. Gi bwok kuom gima nyalo timorene	0	1	2	3	4

YOR WINJRUOK (CHANDRUOK GI….)<br>Be nyathini niga gi chandruok gi	Onge	Machi egni onge	Seche moko	Ndalo duto	Seche tee
1. Winjruok gi nyithindo moko	0	1	2	3	4
2. Nyithindo mok ok dwa bedo osiepege	0	1	2	3	4
3. Ikwinye gi nyithindo moko	0	1	2	3	4

CHAL MAR SKUL(CHANDRUOK GI…)<br>Be nyathini nig a gi chandruok gi…	Onge	Machie gni onge	Seche moko	Ndalo duto	Seche tee
1. Mako gig moko e klas	0	1	2	3	4
2. Wiye wil gi gik moko	0	1	2	3	4
3 Timo tije ge mag skul	0	1	2	3	4

Not to be reproduced without permission
Copyright © 1998 JW Varni, Ph.D.
All rights reserved
