# Supplementary material for: Detection of acute dengue virus infection, with and without concurrent malaria infection, in a cohort of febrile children in Kenya, 2014–2019, by clinicians or machine learning algorithms
Source: PLOS Glob Public Health. 2023 Jul 26;3(7):e0001950. doi: 10.1371/journal.pgph.0001950 (PMC10370704; doi:10.1371/journal.pgph.0001950)
Supplement: Attachment — Submitted filename: Response to Reviewers.doc [file pgph.0001950.s010.doc]

April 28, 2023

Re: PGPH-D-22-02073, “Human or machine? Detection of acute dengue virus infection, with and without concurrent malaria infection, at Kenyan clinics in a cohort of acutely febrile children, 2014-2019”

| **Journal Requirements:** | **Response** |
| --- | --- |
| 1. Please include a complete copy of PLOS’ questionnaire on inclusivity in global research in your revised manuscript. | Done. We have submitted the questionnaire as “S2 Checklist” and have inserted the suggested text in the Methods section (page 3, lines 84-86). |
| 2. Our staff editors have determined that your manuscript is likely within the scope of our Diagnostics in Global Health Call for Papers…If you would like your manuscript to be considered for this collection, please let us know in your cover letter and we will ensure that your paper is treated as if you were responding to this call. | We would like our manuscript to be considered for the collection and have indicated this in the revised cover letter. |
| 3. Please provide separate figure files in .tif or .eps format for Fig 2. | We have uploaded figure files in .tiff format. To avoid confusion, we have excluded the figure files previously submitted in .pdf format. |
| 4. In the online submission form, you indicated that your data will be submitted to the Dryad database upon acceptance. Should your submission be accepted, we will require the following information in your Data Availability Statement:  a. The DOI provided by Dryad  b. The citation for your data package in the reference section of your manuscript  c. The citation for your data package in the methods section | The DOI provided by Dryad is https://doi.org/10.5061/dryad.rn8pk0pg1  We have added the citation to the methods and reference sections. |
| **Reviewer #1 Comments** | **Responses** |
| The presentation of the origin of the data, the organization and the flow of the data should be presented more early and more clearly in the text. The baseline values should appear directly in the text and the differences observed between the study sites should be more discussed. Thus, by further clarifying the context and origin of the data, it will be clearer to understand the specific organization and specific goals of the analysis. | We have added the names of the sites to the beginning of the Methods section (page 4, lines 90-91). We also have expanded the discussion of differences in age, HAZ, BAZ, and wealth observed between the different study sites (page 6, lines 151-158). |
| In general, the authors do not sufficiently present the statistical tools used and/or the statistical results obtained. For example, concerning the clinical manifestations carried out on several categories, when they are significant, are not sufficiently informative: they do not show which value carries the weight of the significant difference (e.g. the contribution of Chi2). In the same way, the results of these tests are insufficiently discussed in the text. | We have created two supplementary tables to provide additional details for the chi square analyses (S2 and S3 Tables), which include residuals and percent contribution. |
| The same remark can be made for the use of the 'misforest' package of R. The authors do not discuss the configurations used and do not present sufficiently the different means used (boosted logistic regression, classification tree, random forest, support vector machines, naive Bayes, neural netork) to evaluate the prediction performances of malaria and dengue… | We have added additional text to explain why we chose the included algorithms (pages 5-6, lines 136-141). The tuning parameters for each model are given in Tables 5 & 6 which summarize model performance. |
| **Reviewer #2 Comments** | **Responses** |
| Prior to publication, the paper should benefit of some clarifications and minimal revision  including for the most important, additional details on machine learning and deep learning methods, better consideration of STROBE recommendations for reporting the findings of observational research. | Our responses to the reviewer’s specific comments follow. We have attached a STROBE statement to the supplementary materials to address the STROBE checklist items highlighted by the reviewer. |
| Optional revision could consist in performing a multinomial logistic regression model aimed at  distinguishing the factors associated with each infection stratum, namely dengue alone, or with  dengue with malaria co-infection, or absence of both conditions, taking malaria alone as referent  stratum (justified by the report that malaria is by far the predominant condition). If the goal is really  to distinguish each stratum, a multinomial logistic regression model is the best way to do, which  minimize the alpha risk, whereas separate logistic regression models carry the risk of identifying  predictors that are not associated when all strata are accounted simultaneously (as in real life). | Thank you for this suggestion. We have updated Table 4 with ORs calculated from the multinomial logistic regression model (pages 16-18), and have updated the methods section (page 5, lines 131-133). |
| 1(b). The title is too long. I would remove “Human or machine” which is not clear and add “using  machine learning” after infection.  Page 1, line 44. “To train” applies for machine learning. Maybe prefer “develop” better appropriate for clinical prediction models.  Page 1, line 46. “To test” applies for machine learning. Maybe prefer “validate” better appropriate for clinical prediction models.  Page 1, lines 48 to 50. “In the Reserved test data” is not explicit. Prefer “In the validation dataset”.  Provide the same indicators for each model (accuracy, sensitivity and specificity) | We have made the suggested changes to the title (page 1) and abstract (page 2). |
| Page 2, line 60 to page 3, line 61. Though the principle of parsimony may be known of a few, it is a philosophical concept that deserves to be explained in a few words for a large majority (e.g, otherwise said...). | Done (page 3, line 62). |
| Page 3, line 69. The term “rubrics” seems informatic jargon. Explain or replace by more explicit term. | Done (page 3, lines 69-70). |
| Hypotheses are lacking. | We have added a statement of our hypothesis to the introduction (page 3, lines 79-81) |
| Page 4, line 81. Be more precise on dates and specify if possible days. | Done (page 3, line 84). |
| Page 4, lines 107 to 109. Dengue diagnosis relies on RT-PCR (gold standard) and IgG antibody  seroconversion. Why not using rapid diagnostic tests as recommended by the WHO ? | Rapid diagnostic tests were unavailable in Kenya during much of the study period. |
| Page 5, lines 116 to 117. Write “Kruskal-Wallis” and not “Kruskall-Wallis”. Simplify the statement and  write “Non parametric Kruskal-Wallis H test and Student T test for continuous data, as appropriate.” | Done (page 5 lines 126-127). |
| Page 5, lines 119 to 120. I don’t understand the justification of setting the Bonferroni correction to  alpha=0.002 given each variable is compared three times (dengue alone vs malaria alone, denguemalaria coinfection vs malaria alone, no dengue no malaria vs malaria alone). | We anticipated comparing the ability of up to 25 clinical features to predict infection class. To aid in selection of features to be used in statistical models, we preset the alpha at 0.002. We applied the same alpha to all comparisons to minimize the risk of false discovery. We have rewritten the text to clarify (page 5, lines 129-132). |
| Page 5, lines 123 to 125. Detail machine learning algorithms in a few words with their particularities,  applications and limits. | Done (page 5-6, lines 136-141) |
| Page 5, line 137. Replace “To train the models” by to “to develop the models.” Replace “was reserved  to test” by “was validated” | Done (page 6, lines 143-144). |
| Provide average delays of presentation to hospital since days of symptom onset for each stratum. | These data were not recorded. |
| Page 7, Table 1. Write “Kruskal-Wallis” and not “Kruskall-Wallis”. | Done (page 7). |
| Page 7, line 156. “Differences in HAZ and BAZ” between which groups ? | We have expanded the text to better explain the differences (page 6, lines 153-156). |
| Page 8, Table 2. Write “Kruskal-Wallis” and not “Kruskall-Wallis”. | Done (page 9). |
| If possible, specify the attrition for each group at time of visit (one month). | We previously counted the 452 subjects lost to follow up as part of the “1273 missing DENV results” in Figure 1. We have now modified Figure 1 to indicate that there were 452 lost to follow up and 821 missing DENV results. We have modified the text to reflect this (page 8, lines 166-168). This did not alter the number of subjects included in the complete cases analysis. |
| Page 10, line 172. Among the 65 variables, there are 23 variables significant at alpha=0.002 and not  28 | In table 3, we count 28 variables that show *p*≤0.002. |
| Page 10, lines 172 to 174. It is unclear how the variables presented in Fig 3 were selected. Explain. | Figure 3 is a visual example of the most commonly encountered symptoms and physical exam findings from Table 3 |
| Figure 3 is heavy loaded and most credibility intervals overlap. I would simplify and use a Venn  Diagram in which I would position each symptom/sign. | We appreciate the reviewer’s comment but are unclear on how to represent the frequencies of individual symptoms among the four groups using a Venn diagram. We reformatted the figure as a bar graph which we hope is easier to read. |
| Specify in table 4 that OR are crude (unadjusted). | We have added this detail to Table 4 (pages 16 – 18). |
| If the purpose here is to distinguish the symptoms  and clinical signs characteristic of each stratum, why not have preferred a multinomial logistic  regression model, instead of performing three models against malaria alone as referent category.  These should be corrected for alpha risk inflation, whereas a multinomial logistic regression allows  discriminating each stratum in one time. | Thank you for this suggestion. We have updated Table 4 with ORs calculated from the multinomial logistic regression model (pages 16-18), and have updated the methods section (page 5, lines 132-133). |
| Page 14, lines 209 to 210. “Only the criterion of rash appeared to be more likely in DENV solo-infected”.  It seems also true for cervical adenopathy. | Cervical adenopathy is not one of the 2009 WHO clinical criteria for diagnosis of dengue. |
| Page 18, lines 253 to 254. “Malaria diagnosis as recorded by clinicians agreed…” Agreement testing with Cohen’s Kappa should be added in the Method section. | Done (page 5, lines 123-124). |
| Table 5. For the clinical diagnosis, there is a discrepancy for Kappa measure between the text and the  table (0.636 or 0.65, respectively). | Thank you for detecting this error. We have corrected Table 5. |
| Add a new table, as Table 7 or eventually as Supplemental table for predicting absence of dengue and absence of malaria stratum. | Done (Table S5). |
| Page 17, line 237. Be more explicit or the ceiling effect. | We have rewritten the text to provide greater clarification (page 18, lines 266-269). |
| Figure 4. Why having not test inter-stratum differences and presented horizontal brackets between  tested columns and stars for setting the p value significance “\|**\|“ | Since *p*≤0.002 for all between strata comparisons, we noted this in the figure legend rather than making the figure busier (page 19, lines 275). |
| Discussion  The discussion is unstructured and resembles to a series of comments. Restructure the  discussion following STROBE guidelines (provide the checklist after first round of revision) | We have reorganized and rewritten the discussion to conform to the order recommended by the STROBE guidelines. |
| Conclusion  Forget Occam and his razor. Focus on take-home messages and perspectives | The large number of DENV/malaria co-infections missed by clinicians demonstrates that Occam’s razor cannot be universally applied. This is an important take-home message. We have changed to terminology to “diagnostic parsimony” (page 25, line 389-390) to match that of the introduction. |
| Discuss the paper entitled “Incidence of chikungunya virus infections among Kenyan children with neurological disease, 2014–2018: A cohort study”, by Niamwaya DK et al (PLoS Med 2022; 19(5): e1003994). as it is an important paper regarding the topic of the study and the study was performed in the same country. | Done (page 24, lines 355-356). Thank you for directing us to this paper. |
| Additional comments  Page 22, line 280. Write “force of infection” and not “force of transmission”. FOI is an epidemiological  parameter  = number of new infections  Number of susceptible exposed x average exposure time | Done (page 24, line 353). |
| All references are not conformed to PGPH standards. Revise. | We have reformatted the bibliography. |
|  |  |
